# Chemotherapy-Related Encephalopathy With Super-Refractory Status Epilepticus in a Child With Osteosarcoma: A Case Report With a Review of Literature

**DOI:** 10.3389/fphar.2019.00963

**Published:** 2019-09-02

**Authors:** Lorenza Di Genova, Katia Perruccio, Maria Grazia Celani, Elena Mastrodicasa, Teresa Anna Cantisani, Susanna Esposito, Maurizio Caniglia

**Affiliations:** ^1^Pediatric Oncohematology Unit, Santa Maria della Misericordia Hospital, Perugia, Italy; ^2^Pediatric Clinic, Department of Surgical and Biomedical Sciences, Università degli Studi di Perugia, Perugia, Italy; ^3^Neurophysiology Unit, Santa Maria della Misericordia Hospital, Perugia, Italy

**Keywords:** cisplatin, encephalopathy, methotrexate, osteosarcoma, psychosis, seizures, status epilepticus

## Abstract

Osteosarcoma is the most frequent primary cancer of the bones, and a combination of primary chemotherapy, surgery, and adjuvant chemotherapy is its current treatment. In adults, some authors have reported problems with memory and concentration following chemotherapy, but in children, severe neurologic dysfunction has been rarely reported. This report describes a 13-year-old patient with primary high-grade nonmetastatic osteosarcoma of the tibia who developed encephalopathy with super-refractory status epilepticus related to chemotherapy. He received methotrexate (MTX) and cisplatin (CDDP)-containing polychemotherapy, and after the first course of drug administration, he developed fever, confusion, a state of psychomotor agitation, and super-refractory status epilepticus with normal laboratory and imaging findings. The causal relationship between the administration of the first polychemotherapy course and his neurological manifestations may be supported by the evaluation and exclusion of other causes. The administration of antiepileptic drugs and off-label atypical antipsychotics was necessary to treat his neurological complications and behavioral changes. This patient represents the first known example of super-refractory status epilepticus in a child treated with MTX and CDDP-containing chemotherapy. Physicians should be aware that encephalopathy and seizures are possible consequences of CDDP therapy when administered alone or in combination with other chemotherapeutic agents. Further studies are needed to better define this relationship in children.

## Background

Osteosarcoma is the most frequent primary cancer of the bones, and its incidence is higher in adolescents and young adults than in other populations (Picci, 2014). Multiagent chemotherapy has been used since the 1970s in patients with osteosarcoma, but no standard chemotherapy regimen has been defined (Hogendoorn et al., 2010). A combination of primary chemotherapy, surgery, and adjuvant chemotherapy is the current treatment strategy for conventional osteosarcoma (ESMO/European Sarcoma Network Working Group, 2014). The therapy recommended in osteosarcoma is represented by doxorubicin (ADM), cisplatin (*cis*-diamminedichloroplatinum [CDDP]), methotrexate (MTX), and ifosfamide (IFO) (ESMO/European Sarcoma Network Working Group, 2014). In the last years, the immunostimulatory drug muramyl tripeptide (MTP-PE) was licensed in Europe for patients < 30 years old after complete surgical recovery (ESMO/European Sarcoma Network Working Group, 2014). The MAP (methotrexate, doxorubicin, and cisplatin) therapy has become the recommended regimen (ESMO/European Sarcoma Network Working Group, 2014).

A poor response to primary chemotherapy has been associated with a negative predictive factor of survival (Ferrari and Serra, 2015). A relationship has also been reported between chemotherapy toxicity and patient prognosis (Ferrari and Serra, 2015). Neurotoxic adverse events occur frequently in chemotherapy and represent a reason to reduce its dose as drugs have been associated with peripheral neurotoxicity, manifesting as peripheral neuropathy and central complications (Verstappen et al., 2003). CDDP is an effective and widely used chemotherapeutic agent that enters only in part the blood–brain barrier, but side effects can occur in the central nervous system (CNS), with encephalopathy, cortical blindness, headache, focal deficits, stroke, and seizures having all been described (Verstappen et al., 2003). Encephalopathy may be caused by a direct toxic effect of CDDP and also by CDDP-induced electrolyte disorders (e.g., hypocalcemia, hypomagnesemia, and hyponatremia because of inappropriate antidiuretic hormone secretion and renal toxicity) (Verstappen et al., 2003). Peripheral complications due to axonal changes secondary to neuronal damage are common adverse effects of CDDP: sensory symptoms usually follow cumulative doses (> 400 mg/kg) (Boogerd et al., 1990; Siegal and Haim, 1990; LoMonaco et al., 1992; van den Bent et al., 2002). CNS symptoms have been associated also with MTX administration. Aseptic meningitis and subacute neurological toxicities consisting of hemiparesis, confusion, ataxia, and seizures occurring 5–10 days have been reported during MTX therapy (Kiu et al., 1994; Genvresse et al., 1999). Moreover, delayed toxicity presenting as leukoencephalopathy is the most devastating complication of MTX and may precede neurological symptoms (Lien et al., 1991; Moore et al., 2002).

Although several studies have explored neurological adverse effects related to chemotherapy in adults, some authors have reported problems with memory and concentration following chemotherapy in children (van Dam et al., 1998; Ahles et al., 2002), but severe neurologic dysfunction has been rarely reported. This report describes a 13-year-old patient with primary high-grade nonmetastatic osteosarcoma who developed encephalopathy with super-refractory status epilepticus related to chemotherapy.

## Case Presentation

A 13-year-old male presented with P-glycoprotein-positive osteoblastic osteosarcoma of the tibia. The patient was treated with MTX (12 g/m^2^), CDDP (120 mg/m^2^), and doxorubicin (DOX; 75 mg/m^2^). Leucovorin rescue treatment (10 mg/m^2^ every 6 h for 12 doses i.v. until MTX level < 0.05 μmol) was included.

Ten days after the end of the first treatment cycle, the patient showed fever, confusion, and psychomotor agitation. Laboratory investigations revealed neutropenia (0.58 × 10⁹/L) and increased levels of C-reactive protein (CRP). Serum calcium, potassium, and magnesium levels were normal. The next day, a neurological examination showed an acute confusional state, stereotypical movements of the lower limbs, head turning movements, fatuous smiling, echolalia, and impaired consciousness. Magnetic resonance imaging (MRI) and cerebrospinal fluid (CSF) examination gave normal results; in particular, a CSF exam documented normal cell counts and protein and glucose values. Antibodies against onconeural and/or neuronal cell-surface antigens were not detected in the serum and/or CSF. Electroencephalography (EEG) revealed frontal status epilepticus (SE). SE persisted despite i.v. lorazepam (0.05 mg/kg) and i.v. phenytoin (15 mg/kg); oral oxcarbazepine (60 mg/kg/day) was started. He was given high continuous i.v. midazolam (0.06 mg/kg/h), which produced good clinical and electrical improvement. Nevertheless, SE recurred on midazolam weaning. Therefore, in addition to oral oxcarbazepine, he was given oral high-dose lorazepam (0.15 mg/kg/day) with the aim of switching from parenteral to oral administration of benzodiazepine. In addition, he presented a psychotic status characterized by agitation and disinhibition for which oral risperidone (0.25 mg/day) was administered.

Over the next days, he gradually improved, and after a week, no seizures occurred, and no paroxysmal discharges were observed on EEGs. Repeated neurological examinations, including MRI, EEG, and CSF examinations, documented no new findings; in particular, there was no evidence of paraneoplastic syndrome, metastases, or cerebrovascular complications. His mental status improved with the resolution of psychotic symptoms. He received other courses of MTX-containing polychemotherapy (not including CDDP) without complications.

After 3 months, his osteosarcoma was treated with surgical resection. In addition, he achieved a very good response (post-chemotherapy necrosis grade: 99%) and was treated with further courses of low-dose CDDP (80 mg/m^2^) and MTX (8 and 10 g/m^2^) with no further seizures. He is currently on therapy with oral high-dose lorazepam, oxcarbazepine, and risperidone with control of neurologic and psychotic symptoms.

The management of this patient was approved by the Ethics Committee of Umbria Region (PED-2018-002), and both parents provided written informed consent for the exams and therapy of the child. The Ethics Committee of Umbria Region approved the publication of this case, and both parents provided written informed consent for the publication of this manuscript.

## Discussion

In this case study, we describe the unusual presentation of a young patient with super-refractory SE attributed to MTX and CDDP-containing chemotherapy. The patient had impaired consciousness, psychomotor agitation, focal seizures, and EEG results that showed frontal SE with normal laboratory and imaging findings. A causal relationship between the administration of the first polychemotherapy course and his neurological manifestations may be supported by the evaluation and exclusion of other causes, such as metastasis, cerebrovascular accident, venous thrombosis, paraneoplastic syndromes, or infective complications.

The administration of antiepileptic drugs was necessary to treat his neurological complications: the initial therapy, based on phenytoin and lorazepam intravenous bolus administration followed by midazolam infusion, was useful in arresting his super-refractory SE. The combination of oral lorazepam and oxcarbazepine was effective, as no further seizures were observed, and EEGs became normal. Our patient experienced also behavioral changes characterized by an excessive sense of foreboding and agitation. Therefore, he was given off-label atypical antipsychotics, such as risperidone, which produced significant improvement in his mental status. He received further courses of MTX and CDDP-containing chemotherapy based on a low dose of CDDP administration without complications.

Intrathecal MTX administration has been reported to induce aseptic meningitis in 40% of young patients (Geiser et al., 1974). Moreover, chronic encephalopathy associated with confusion, ataxia, and dementia has been described in pediatric patients with acute lymphoblastic leukemia who received long-term low-dose intravenous MTX, associated with intermittent intrathecal MTX therapy (Kay et al., 1972). Patients with brain tumors treated with intraventricular MTX could also develop leukoencephalopathy (Sullivan et al., 1969). Allen et al. described an unusual neurological syndrome in four out of 158 patients treated for osteogenic sarcoma following a chemotherapy regimen that included vincristine and methotrexate (Allen and Rosen, 1978). The average age of the patients was 18.6 years, and three males and one female were affected. The doses of MTX were comparable among the four patients and ranged from 8 to 9 g/m^2^. The average interval between MTX administration and the occurrence of encephalopathy was 10 days. The onset of neurological symptoms was usually abrupt. The syndrome clinically appeared as a cerebrovascular accident with signs of focal cerebral dysfunction and fluctuated over several days. Two patients experienced behavioral changes. Clinical manifestations usually disappeared within 72 h. Although we can draw some analogies with our clinical case, neurologic symptoms in our patient appear different from those previously attributed to MTX therapy alone. In our patient, cerebral MRI was persistently normal and showed no signs of embolic cerebral vasculopathy or cerebral atrophy. Therefore, it is important to analyze neurotoxicity that may be induced by the combination of MTX and CDDP chemotherapy.

Two types of CNS complications have been associated with CDDP exposure. The first is represented by posterior leukoencephalopathy syndrome (PRES), which consists in cortical blindness, a decreased level of consciousness, seizures, and hypertension (Highley et al., 1992). This complication can occur at the end of CDDP chemotherapy; despite the severity of this clinical syndrome, the majority of the patients make a full recovery (Steeghs et al., 2003; Zahir et al., 2012; Kabre and Kamble, 2016). Focal neurological deficits or a decreased level of consciousness, with or without seizures, has been described (Berman and Mann, 1980; Mead et al., 1982; Gorman et al., 1989; Brauers et al., 1997; Fuse-Nagase et al., 1997; Chue et al., 2009; Rohitashwa et al., 2016). Holman et al. reported a case of SE associated with CDDP in a 54-year-old patient with recurrent cervical carcinoma without other underlying medical conditions (Holman et al., 2015). The patient developed altered mental status at the beginning of the sixth cycle of topotecan and CDDP-containing chemotherapy. The authors recommended the use of alternative chemotherapeutic regimens when platinum-based chemotherapy is suspected as the etiology of neurological complications (Holman et al., 2015). Philip et al. described a 59-year-old woman with metastatic adenocarcinoma of unknown origin; this patient was treated with hydroxyurea and CDDP (75 mg/kg) every 3 weeks (Philip et al., 1991). She presented confusion and then SE 4 days after the third cycle of therapy. The authors highlighted the importance of including CDDP-induced seizures and cortical blindness in the differential diagnosis of focal cerebral signs in patients treated with CDDP (Philip et al., 1991).

Very few studies have explored the relationship between neurotoxicity and CDDP administration in children (**Table 1**). Brock et al. retrospectively studied tolerance to and the toxicity of CDDP treatment in 30 infants (Brock et al., 1992). All children diagnosed under the age of 1 year who received CDDP as part of multiagent chemotherapy and were administered the first dose before their first birthday were eligible for the study. Seizures occurred in two infants; however, in both of these cases, there were other electrolyte disturbances, such as severe hyponatremia, hypocalcemia, and hypomagnesemia (Brock et al., 1992). Highley et al. reported that four of eight children aged 4, 6, 7, and 9 years who had advanced neuroblastoma and were managed with a rapid-delivery high-dose intensity CDDP-based regimen developed acute neurological toxicity (Highley et al., 1992). The authors highlighted that seizures and cortical dysfunction are mainly associated with high doses of CDDP (40 mg/kg/day for 5 days) and in the presence of renal impairment, hypomagnesemia, and febrile neutropenia. They also suggested that a potential mechanism of CDDP-induced seizures was that they are secondary to an unidentified insult to the cerebral vasculature. Gorman et al. described four patients with focal encephalopathy that occurred after CDDP treatment (Gorman et al., 1989). The fourth case was a 13-year-old girl with dysgerminoma of the left ovary; the patient was treated with CDDP–vinblastine–bleomycin and developed focal seizures and postictal left-sided paresis. Normal function returned within 1 h. Investigations showed an elevated serum creatinine level in addition to hyponatremia and neutropenia. A computed tomography brain scan showed no abnormality, and no EEG was performed. Six hours after her admission to the hospital, she became febrile and was treated with antibiotics. She received two further courses of CDDP–vinblastine–bleomycin without complications. In each of these four cases, the focal encephalopathy completely resolved.

**Table 1:** Cisplatin-induced encephalopathy with seizures reported in children and adolescents.

Paper	Tumor type	Patients (age/sex)	Co-treatment	Encephalopathy	Seizures	Therapeutic approach to seizures	Starting time	EEG	Brain imaging: MRI/CT	Electrolytes
Brock et al. (1992)	NR	2 infants (<1 year old)	NR	NR	SNS	NR	NR	NR	NR	Hypocalcemia, severe hyponatremia, and hypomagnesemia
Highley et al. (1992)	Neuroblastoma	1. 9/F 2. 7/F 3. 6/F 4. 4/F	VCR, VP-16, and Cl	1. NR 2. NR 3. NR 4. Headache, visual loss, nystagmus on the left	1. GMS 2. GMS 3. AS 4. NR	1. DZP and PHB → effective 2. PHT and CBZ → effective 3. Prophylactic CBZ → not effective 4. Prophylactic CBZ → not effective	1. Day 8 after the 4th course 2. Day 3 after the 1st course 3. Day 5 after the 4th course 4. Day 17 after the 6th course	1. NP 2. Normal 3. Marked bilateral abnormality, particularly over the posterior head region	1. Normal 2. Normal 3. Multiple low-density lesions 4. Sagittal sinus thrombosis	1. Hypokalemia, hyponatremia, and hypomagnesemia 2. Hyponatremia 3. Hypokalemia and hypomagnesemia 4. Hypokalemia and hypomagnesemia
Gorman et al. (1989)	1. Metastatic embryonic cell carcinoma 2. Embryonic cell carcinoma and an undifferentiated teratoma 3. Seminoma 4. Dysgerminoma of the left ovary	1. 32/M 2. 26/M 3. 32/M 4. 13/F	1. VBL and BLEO 2. VBL and BLEO 3. VBL 4. VBL and BLEO	1. Loss of vision in both eyes 2. Expressive aphasia 3. Aphasia, right homonymous hemianopia 4. NR	1. NR 2. FS 3. NR 4. FS	1. NR 2. DZP → effective 3. NR 4. NR	1. 6 days after the 5th course 2. 13 days after the 3rd course 3. 10 days after the 7th course 4. 11 days after the 2nd course	1. NR 2. NR 3. Slow-wave activity over the left hemisphere of the brain 4. NP	1. Normal 2. Normal 3. Normal 4. Normal	1. Hypomagnesemia 2. Hypomagnesemia 3. Hypomagnesemia 4. Normal

AS, absence of seizures; BLEO, bleomycin; CBZ, carbamazepine; Cl, chlorambucil; DZP, diazepam; FS, focal seizure; GMS, grand mal seizure; MZL, midazolam; NP, not performed; NR, not reported; PHB, phenobarbital; PHT, phenytoin; SE, status epilepticus; SNS, seizure not specified; VBL, vinblastine; VCR, vincristine; VP-16, etoposide.

## Conclusions

Our case is the first known example of super-refractory SE in a child treated with MTX and CDDP-containing chemotherapy. Physicians should be aware that encephalopathy and seizures are possible consequences of CDDP therapy when administered alone or in association with other chemotherapeutic agents. Further studies are needed to better define this relationship in children. This case report indicates that careful clinical monitoring must be performed in young patients receiving systemic polychemotherapy, and it should be taken into consideration that CDDP lowers the seizure threshold. Definitive precipitating factors have not been established, although our experience suggests that febrile neutropenia may be an associated condition. Other possible conditions that can facilitate these rare neurological complications include renal impairment and dyselectrolytemia. Fortunately, encephalopathy after CDDP chemotherapy appears to be reversible and, in our experience, may not necessarily preclude further treatment with the drug. Future studies should clarify the best strategies to manage encephalopathy with SE in children who are treated with chemotherapy and later develop these rare neurological complications.

## Data Availability

All datasets generated for this study are included in the manuscript/supplementary files.

## Ethics Statement

This case report was approved by the Ethics Committee of Umbria Region (PED-2018-002), and both parents gave written informed consent for the evaluation of themselves and the child.

## Author Contributions

LDG and KP wrote the first draft of the manuscript in charge of the patient's follow-up. MGC and TAC took care of neurologic management. EM was in charge of the patient's follow-up. MC supervised patient's management and gave a substantial scientific contribution, SE gave scientific contribution and critically revised the paper. All the authors have read and approved the final version of the manuscrift.

## Funding

This study was partially supported by a grant from the Pediatric Section, Department of Surgical and Biomedical Sciences, University of Perugia, Perugia, Italy (PED 2019_01).

## Conflict of Interest Statement

The authors declare that the research was conducted in the absence of any commercial or financial relationships that could be construed as a potential conflict of interest.
